# *O*-2-Alkylated Cytosine Acyclic Nucleoside Phosphonamidate Prodrugs Display Pan-Genotype Antiviral Activity against African Swine Fever Virus

**DOI:** 10.1128/msphere.00378-22

**Published:** 2022-11-01

**Authors:** Leah V. Goulding, Eleonóra Kiss, Robert Vrancken, Nesya Goris, Min Luo, Elisabetta Groaz, Piet Herdewijn, Linda Dixon

**Affiliations:** a The Pirbright Institute, Pirbright, Woking, United Kingdom; b ViroVet NV, Leuven, Belgium; c KU Leuven, Rega Institute for Medical Research, Medicinal Chemistry, Leuven, Belgium; University of Texas Southwestern Medical Center

**Keywords:** African swine fever virus, DNA virus, antiviral agents, veterinary pathogens

## Abstract

African swine fever virus (ASFV) causes a highly contagious hemorrhagic disease with case fatality rates approaching 100% in domestic pigs. ASFV is responsible for substantial economic losses, but despite ongoing efforts, no vaccine or antiviral agent is currently available. Attempts to control the spread of ASFV are dependent on early detection, adherence to biosecurity measures, and culling of infected herds. However, an effective antiviral agent may be used in lieu of or in conjunction with a vaccine to effectively curb ASFV outbreaks. The dose-dependent antiviral activities of two amidate prodrugs (compounds 1a and 1b) of *O*-2-alkylated 3-fluoro-2-(phosphonomethoxy)propyl cytosine [(*R*)-*O*-2-alkylated FPMPC] against ASFV isolates of four different genotypes were determined. Both compounds were found to inhibit ASFV progeny virus output by >90% at noncytotoxic concentrations (<25 μM) in primary porcine macrophages. Analysis of viral transcription and viral protein synthesis indicated that these acyclic nucleotide analogues inhibited late gene expression. Interestingly, time-of-addition studies suggest different viral targets of the compounds, which may be attributed to their differing amino acid prodrug moieties. In view of their promising antiviral activity, these nucleotide analogues merit further evaluation as potential prophylactic and/or therapeutic agents against ASFV infection and their antiviral efficacy *in vivo* should be considered.

**IMPORTANCE** African swine fever virus is a highly contagious hemorrhagic viral disease. Since its transcontinental spread to Georgia in 2007, ASFV has continued to spread across the globe into countries previously without infection. It is responsible for substantial losses in the domestic pig population and presents a significant threat to the global swine industry. Despite ongoing efforts, there are no vaccines currently available; in their absence, antiviral agents may be a viable alternative. The significance of our research is in identifying the pan-genotype antiviral activity of prodrugs of *O*-2-alkylated 3-fluoro-2-(phosphonomethoxy)propyl cytosine, which will drive further research on the development of these compounds as antivirals against ASFV.

## INTRODUCTION

African swine fever (ASF) is a highly infectious hemorrhagic disease that causes fatality approaching 100% in domestic pigs and wild boar and is a major threat to the global swine industry (1, 2). Clinical signs of ASF include increased temperature, lethargy, loss of appetite, respiratory distress, vomiting, and abortion in pregnant sows (3). The causative agent of ASF, African swine fever virus (ASFV), is a large DNA virus of the *Asfarviridae* family (4). It has a double-stranded DNA genome of 170 to 190 kb containing 151 to 167 open reading frames (ORFs) (5, 6). ASFV is endemic to Africa, where it was first identified in Kenya in 1921, and it has been identified in up to 35 African countries (7, 8). Since its emergence in the Republic of Georgia in 2007, ASF has spread throughout Europe and the Russian Federation (9, 10). In 2018, ASF was first reported in China, which is responsible for almost half of the world’s pork production, and it has continued to spread throughout Asia (11, 12). Within the first year of the ASF outbreaks in China, there was an estimated loss of 43.46 million pigs, and the total economic loss has been estimated to reach US$111 billion (13). In Poland, there has been a significant decline in the number of pig farms attributed to the ASF epidemic, with a reduction from 260,000 farms in 2012 to approximately 124,500 farms at the end of July 2019. Furthermore, the cost of controlling ASFV within Poland, including the biosecurity and ASF early detection programs, was approximately €97.6 million between 2014 and 2020 (14). Modeling of the economic impact of an outbreak of ASF in Denmark, based on the swine population, movement, and control strategies in Danish swine herds, predicted that the total economic loss of an ASF epidemic could range between €256 million and €442 million (15).

Despite the economic and animal welfare implications of the continued spread of ASF, there are no approved vaccines or antiviral agents available. Current measures to control ASF depend on rapid identification of the disease and strict adherence to biosecurity measures (16). While vaccine development remains a key focus of current research, antiviral agents are a potential viable strategy for controlling ASFV outbreaks (17, 18). Different stages of the ASFV replication cycle have been targeted for antiviral development, such as inhibition of viral genome replication or inhibition of viral proteases. Nucleotide analogue (*S*)-9-(3-hydroxy-2-phosphonylmethoxypropyl)adenine [(*S*)-HPMPA] and its derivatives were found to inhibit ASFV replication *in vitro* by inhibiting viral DNA synthesis and late viral protein expression (19, 20). Alternatively, genistein and fluoroquinolones, such as enrofloxacin, were found to impair viral genome replication by inhibiting the ASFV type II topoisomerase (21–23). Viral protease inhibitors OPTX-1 and E-64 inhibit the activity of S273R, a highly conserved ASFV protease of the SUMO-1 cysteine protease family, impairing ASFV replication (24, 25). Other inhibitors of ASFV replication have also been described but the viral or cellular targets have not been fully defined. These include apigenin, which is proposed to inhibit the early stages of ASFV replication, and resveratrol and oxyresveratrol, which inhibited the later stages of ASFV replication (26, 27).

Currently, 24 genotypes of ASFV have been identified (28), and a broad-spectrum antiviral against multiple ASFV genotypes would be greatly beneficial. Here, we report on two amidate prodrugs (compounds 1a and 1b) of an *O*-2-alkylated 3-fluoro-2-(phosphonomethoxy)propyl acyclic nucleoside phosphonate derivative featuring cytosine as a nucleobase [(*R*)-*O*-2-alkylated FPMPC], which exhibited broad-spectrum antiviral activity in porcine primary bone marrow-derived macrophages at noncytotoxic concentrations against ASFV field isolates of four genotypes.

## RESULTS

### Amidate prodrugs of (*R*)-*O*-2-alkylated FPMPC inhibit ASFV replication in primary porcine bone marrow-derived macrophages at noncytotoxic concentrations.

**FIG 1:**
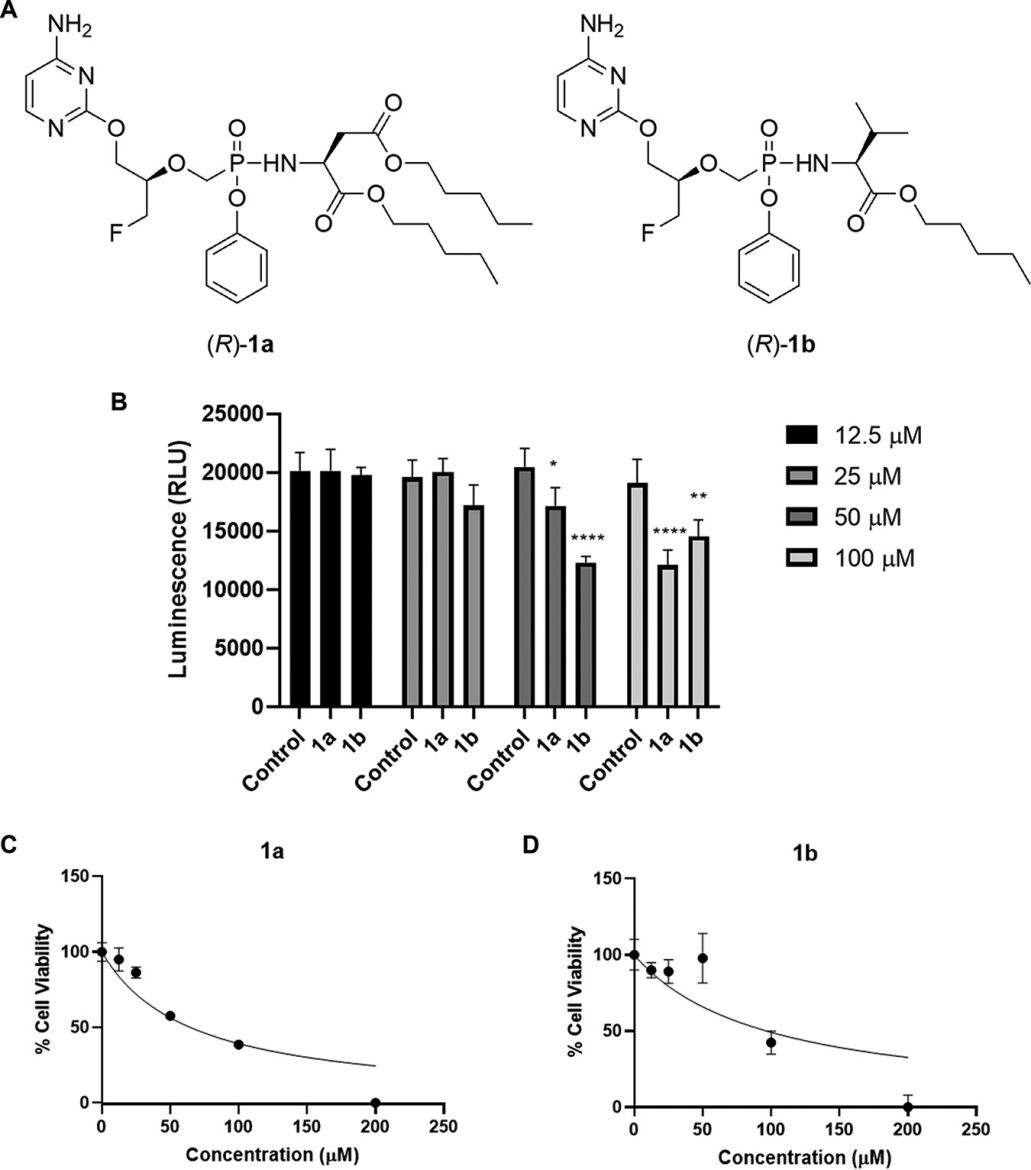
Cytotoxicity of extended treatment of compounds 1a and 1b in primary cells. (A) Chemical structure of acyclic nucleoside phosphonamidate prodrugs 1a and 1b evaluated in this study. (B to D) Purified PBMs were treated with compound 1a or 1b at the indicated concentrations and cell viability, indicated by RealTime-Glo MT cell viability assay, was measured 72 h later. (B) Cell viability following treatment of cells with the compounds relative to the corresponding control (DMSO). Significance was determined by two-way ANOVA, relative to the corresponding control. (C and D) The CC_50_ was calculated from relative cell viability (MT live cell viability assay) by nonlinear regression. Graphs are representative of at least three independent experiments; values represent means and standard deviations. *, *P* ≤ 0.05; **, *P* ≤ 0.01; ***, *P* ≤ 0.001; ****, *P* ≤ 0.0001.

In a previous study, we established a reliable synthetic route for the preparation of chiral *O*-alkylated acyclic nucleoside phosphonates featuring a fluorinated aliphatic pseudosugar side chain linked to the 2-*O* position of a pyrimidine base (e.g., cytosine) in place of the most common nitrogen atom at position 1 [as occurring for instance in the case of (*S*)-HPMPA] (29). Compounds 1a and 1b, containing an l-aspartic acid diamyl ester and l-valine amyl ester phenoxyamidate group as phosphonate prodrug moieties, respectively, were readily obtained from their parent phosphonate (*R*)-*O*-2-alkylated FPMPC using standard conditions for nucleoside phosphonate prodrug derivatization (Fig. 1A). Both amidate prodrugs were found to possess micromolar inhibitory activity against a broad range of human DNA viruses, including human cytomegalovirus (HCMV), varicella-zoster virus (VZV), and hepatitis B virus (HBV).

To determine the potential of compounds 1a and 1b as antiviral agents against ASFV, cytotoxicity was first evaluated in purified pig bone marrow cells (PBMs) exposed to either compound 1a or 1b at different concentrations for 72 h. Cell viability was measured using the RealTime-Glo MT cell viability assay. Incubation with compound 1a or 1b for 72 h at 25 μM and below was not associated with a significant reduction in cell viability (Fig. 1B). Compounds 1a and 1b displayed 50% cytotoxic concentrations (CC_50_) of 75.2 and 63.2 μM, respectively (Fig. 1C and D).

**FIG 2:**
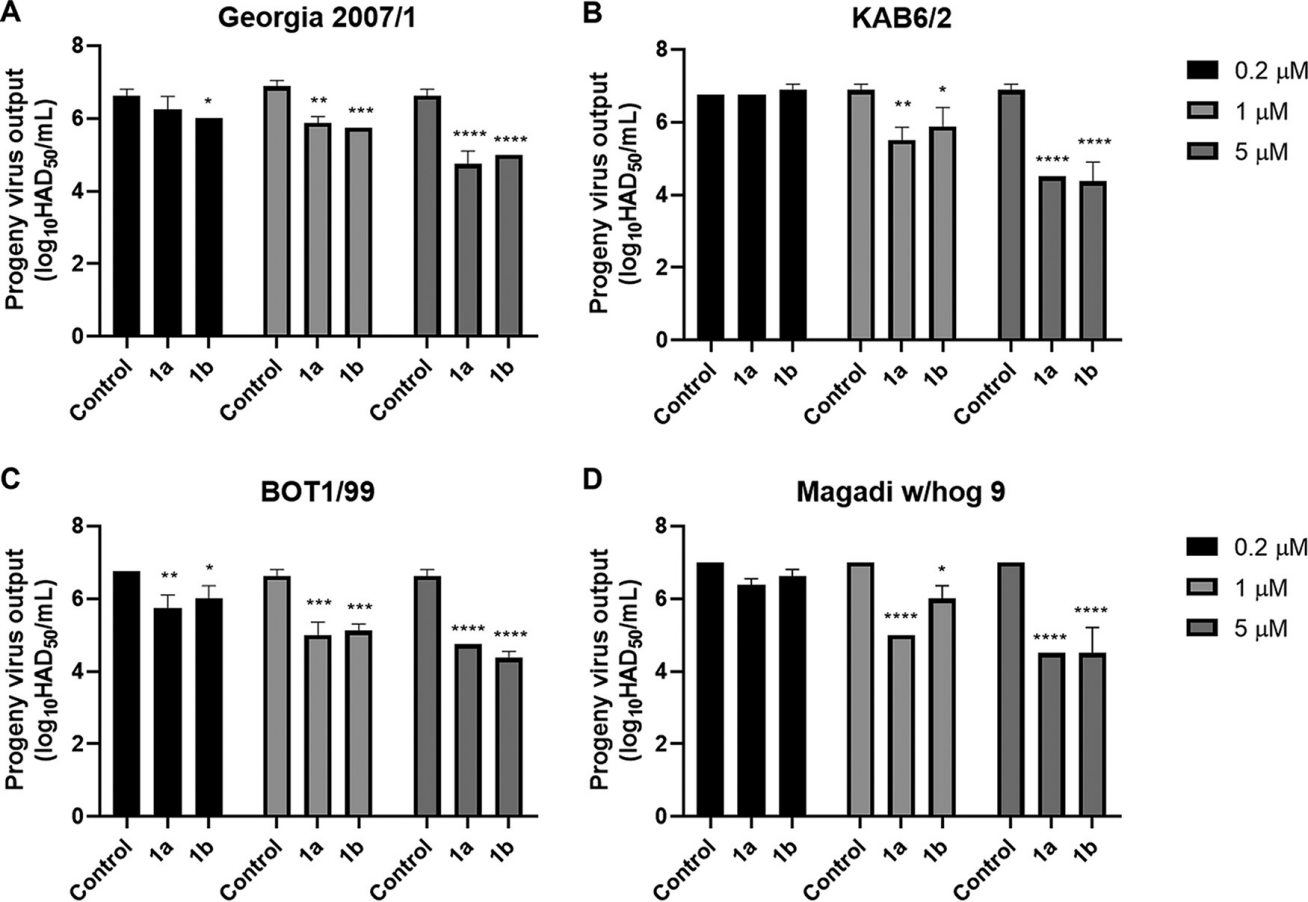
Phosphonamidate nucleoside prodrugs 1a and 1b inhibit ASFV replication in primary cells. Purified PBMs were treated with compound 1a or 1b at the indicated concentrations and infected with Georgia 2007/1 (A), KAB6/2 (B), BOT 1/99 (C), and Magadi w/hog 9 (D) at an MOI of 0.1 for 72 h. Progeny virus output determined by hemadsorption assay, presented as log_10_ HAD_50_ per milliliter. Graphs are representative of three independent experiments; values represent means and standard deviations. Significance was determined by two-way ANOVA, relative to the corresponding control. *, *P* ≤ 0.05; **, *P* ≤ 0.01; ***, *P* ≤ 0.001; ****, *P* ≤ 0.0001.

Subsequently, we determined the antiviral activity of compounds 1a and 1b against four genotypes of ASFV (Georgia 2007/1, KAB6/2, BOT 1/99, and Magadi w/hog 9) in purified PBMs. The reduction in progeny virus output in the supernatant was first measured by hemadsorption assay following a 72-h infection at a multiplicity of infection (MOI) of 0.1. Both compounds 1a and 1b reduced progeny virus output, expressed as 50% haemadsorbing doses per milliliter and presented as log_10_ HAD_50_ per milliliter, by over 95% for all genotypes at 5 μM. Compound 1a displayed greater inhibitory activity than compound 1b at lower concentrations. At 1 μM, compound 1a reduced progeny virus output by ~90% for all genotypes, while compound 1b at 1 μM reduced BOT 1/99 by >90%, Magadi w/hog 9 and Georgia 2007/1 progeny virus output by 85%, and KAB6/2 by ~80% (Fig. 2).

**FIG 3:**
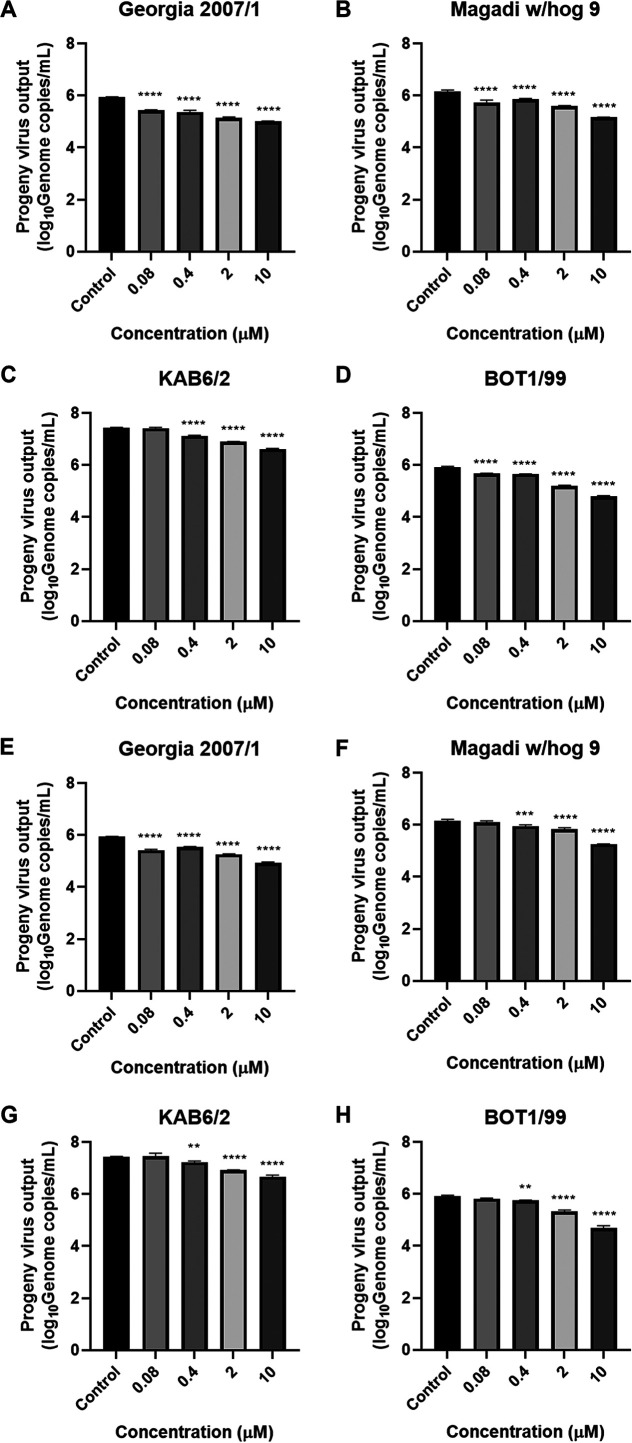
Dose-dependent inhibition of ASFV progeny virus output by compounds 1a and 1b. Purified PBMs were treated with compound 1a (A to D) or 1b (E to H) at the indicated concentrations and infected with Georgia 2007/1 (A and E), Magadi w/hog 9 (B and F), KAB6/2 (C and G), or BOT 1/99 (D and H) at an MOI of 0.5. Supernatants were collected 24 h after infection for viral DNA extraction. Genome copies in the supernatant were determined by VP72 genome copy numbers, relative to a VP72 plasmid standard curve, and presented as log_10_ genome copies per milliliter. Graphs are representative of 3 independent experiments; values represent means and standard deviations. Significance was determined by one-way ANOVA, relative to the DMSO control. *, *P* ≤ 0.05; **, *P* ≤ 0.01; ***, *P* ≤ 0.001; ****, *P* ≤ 0.0001.

Following infection at an MOI of 0.5 for 24 h, levels of the virus genome were detected in cell supernatants by real-time quantitative PCR (qPCR). Similarly, a dose-dependent reduction in progeny virus output in the supernatant was observed for both compound 1a (Fig. 3A to D) and compound 1b (Fig. 3E to H) for each of the four virus genotypes examined, on average reducing progeny virus output by 1 log (90% inhibition) following treatment with compound 1a or 1b at 10 μM.

**FIG 4:**
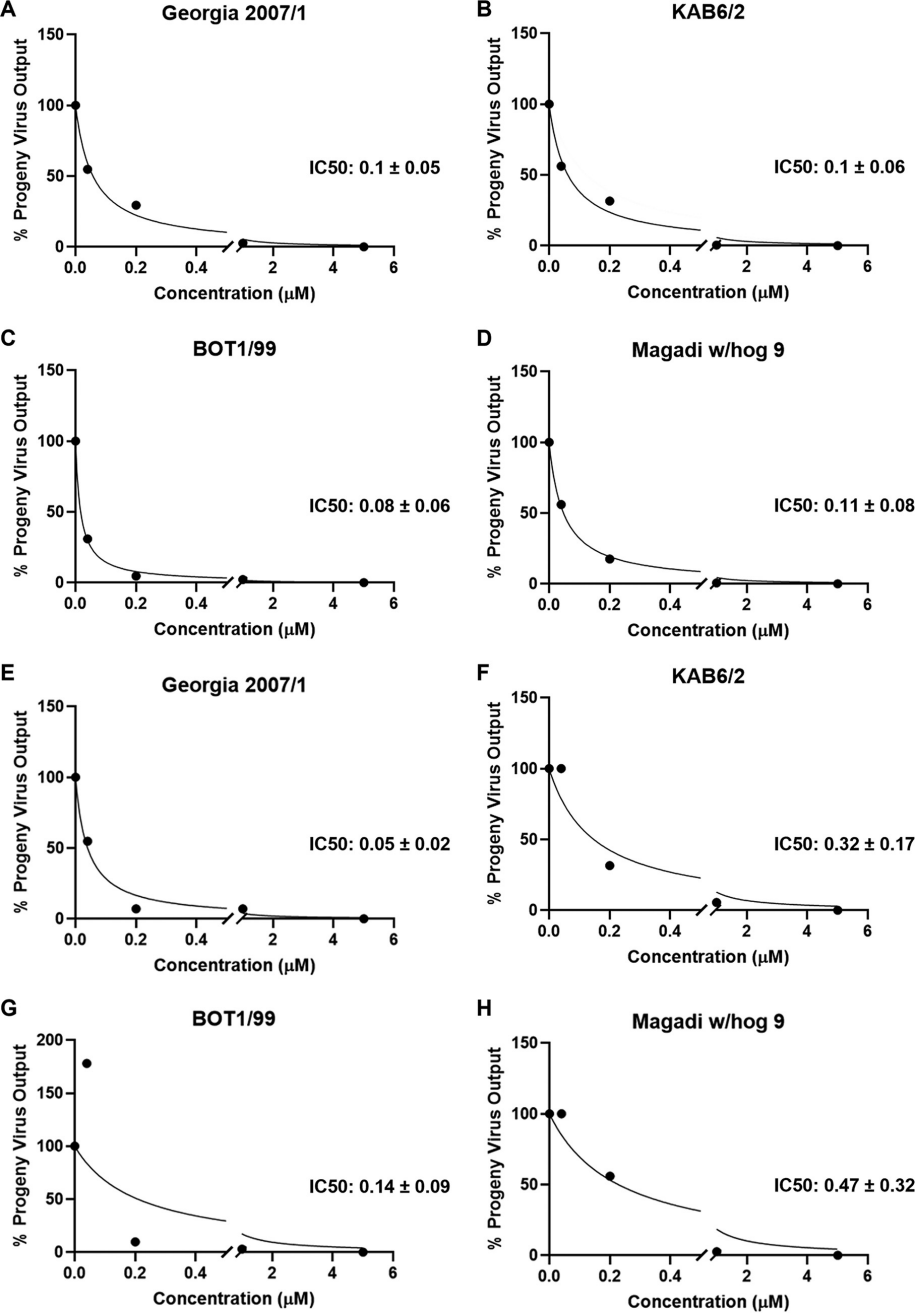
IC_50_ for phosphonamidate nucleoside prodrugs 1a and 1b. Purified PBMs were treated with compound 1a (A to D) or 1b (E to H) and infected with Georgia 2007/1 (A and E), KAB6/2 (B and F), BOT 1/99 (C and G), and Magadi w/hog 9 (D and H) at an MOI of 0.1 for 72 h. The progeny virus output compared to that of control mock-treated cells was determined by hemadsorption assay. The IC_50_ value was calculated by nonlinear regression. Graphs are representative of three independent experiments.

The 50% inhibitory concentrations (IC_50_) were calculated from progeny virus output from a 72-h infection. Overall, the IC_50_s ranged between 0.1 and 0.11 μM for compound 1a and 0.05 and 0.47 μM for compound 1b (Fig. 4). The selectivity indices (SI; CC_50_/IC_50_) of compound 1a against Georgia 2007/1, KAB6/2, BOT 1/99, and Magadi w/hog 9 were 744.8, 752.5, 940.6, and 648.05, respectively. Correspondingly, compound 1b selectivity indices were 1,194, 197.5, 451.5, and 134.5. Overall, these data demonstrate that both compounds 1a and 1b display potent antiviral activity against multiple wild-type ASFV genotypes at noncytotoxic concentrations.

### Amidate prodrugs of (*R*)-*O*-2-alkylated FPMPC inhibit transcription of late ASFV genes.

**FIG 5:**
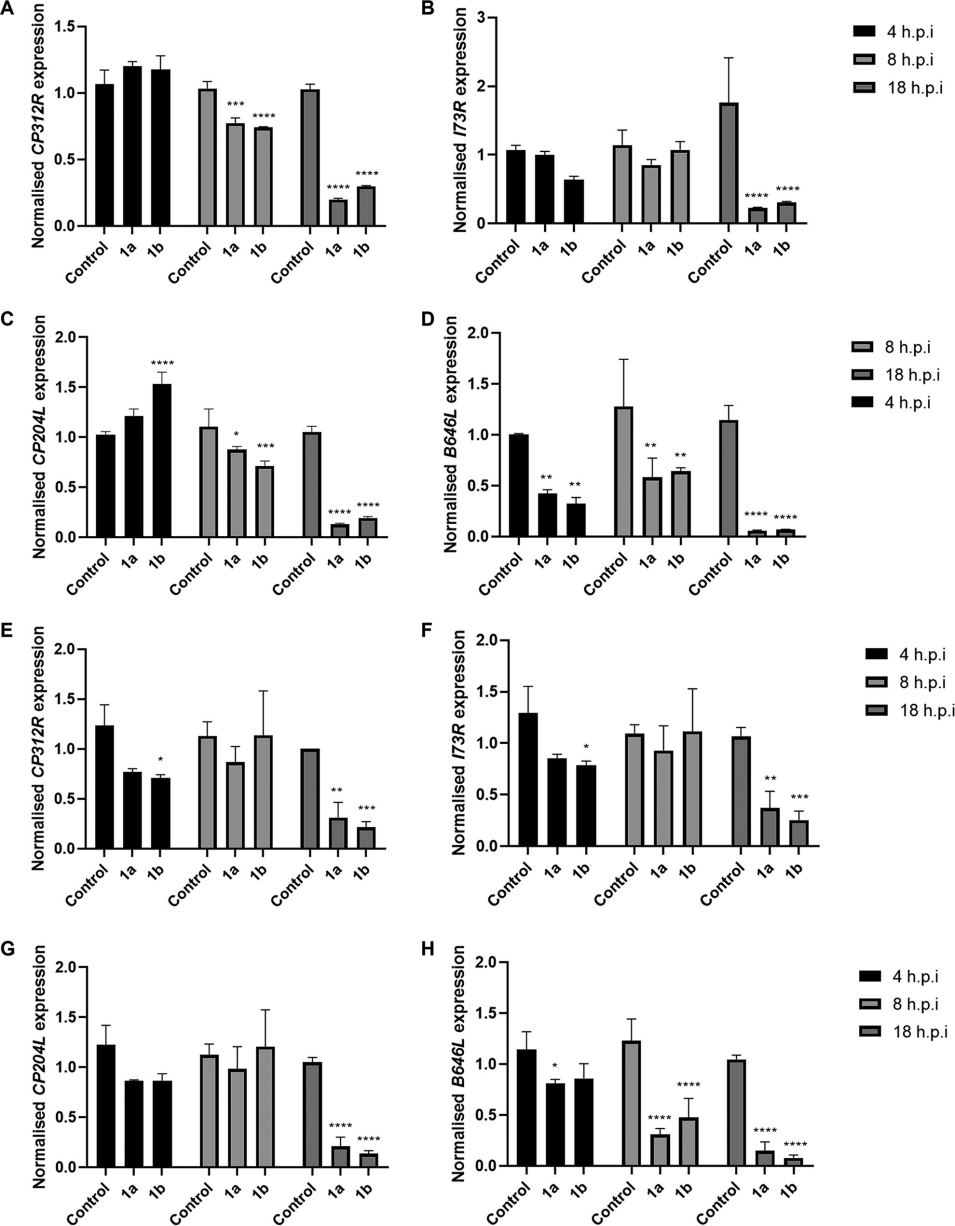
Reduced late gene expression in amidate prodrug-treated cells infected with wild-type ASFV. Purified PBMs were treated with the indicated compound at 10 μM and infected with Georgia 2007/1 (A to D) or BOT 1/99 (E to H) at an MOI of 1.0. Total RNA was extracted at 4, 8, and 18 h postinfection. *CP204L*, *CP312R*, *I73R*, and *B646L* transcript levels, normalized to *GAPDH* RNA, were determined relative to the corresponding control. Graphs are representative of 3 independent experiments; values represent means and standard deviations. Significance was determined by two-way ANOVA, relative to the corresponding control. *, *P* ≤ 0.05; **, *P* ≤ 0.01; ***, *P* ≤ 0.001; ****, *P* ≤ 0.0001.

ASFV gene expression is temporally regulated and follows a cascade pattern of transcription. The viral transcripts may be divided into categories according to their time of expression within the replication cycle. Expression of immediate and early transcripts occurs before DNA replication, while expression of intermediate and late transcripts is dependent on ASFV genome replication (30). To determine if compounds 1a and 1b inhibited ASFV replication before or after DNA replication, we assessed the levels of transcripts for early and late genes in purified PBMs treated with the amidate prodrugs and infected with either Georgia 2007/1 or BOT 1/99 at an MOI of 1.0. Early viral gene *CP312R* and *I73R*, early and late gene *CP204L*, and late gene *B646L* transcript levels were measured at 4, 8, and 18 h postinfection. Early gene transcripts were not consistently downregulated at 4 and 8 h postinfection in cells infected with either virus, but levels were significantly reduced relative to the control at 18 h postinfection (Fig. 5A to C and E to G). Additionally, we consistently observed a significant downregulation of *B646L* transcript levels at 8 and 18 h postinfection in purified PBMs infected with either Georgia 2007/1 or BOT 1/99 (Fig. 5D and H).

### Amidate prodrugs of (*R*)-*O*-2-alkylated FPMPC inhibit late virus protein expression.

**FIG 6:**
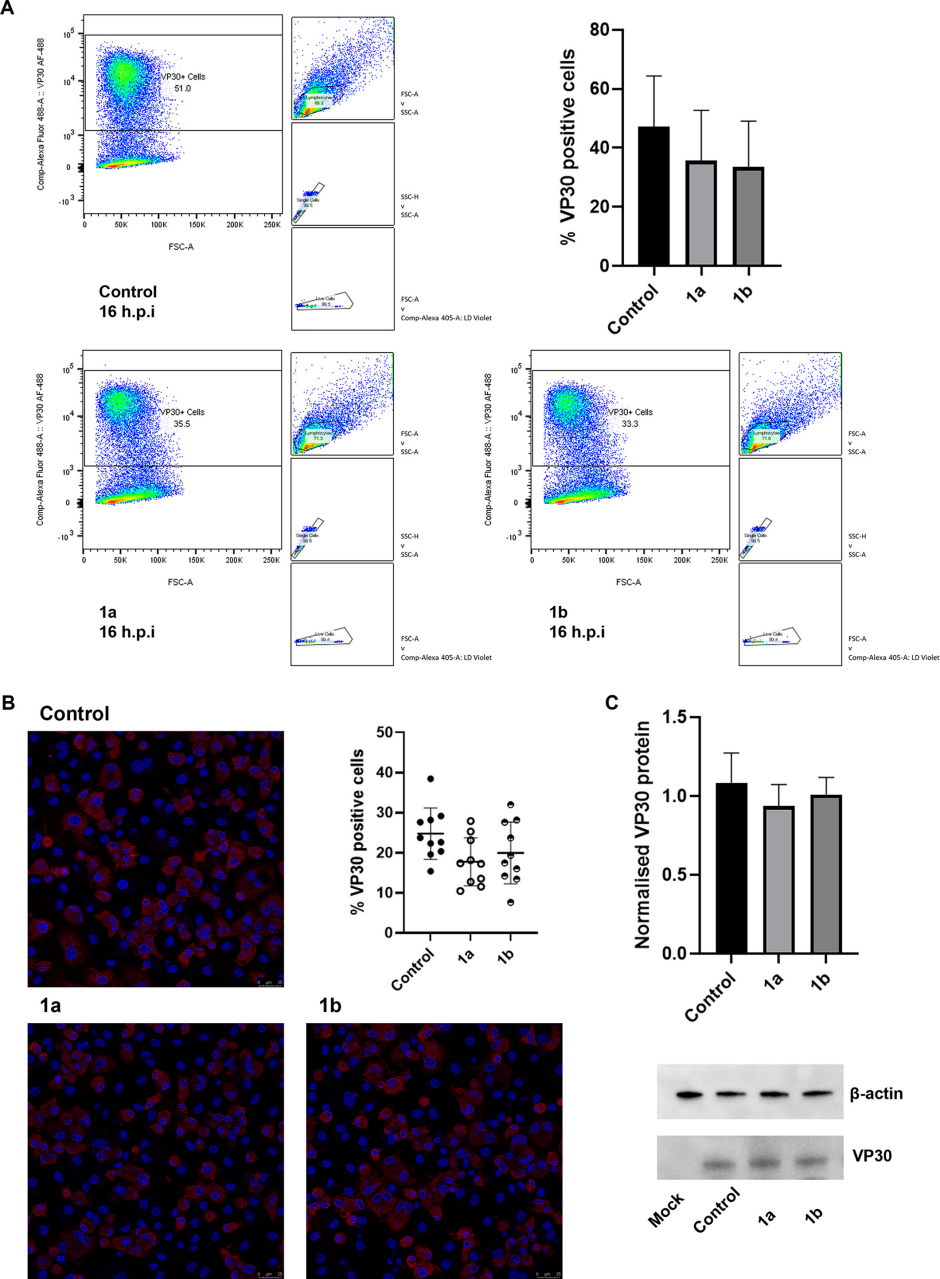
No significant reduction in early protein expression following treatment with compound 1a or 1b. (A) Purified PBMs were treated with compound 1a or 1b at 10 μM or DMSO and infected with Georgia 2007/1 at an MOI of 0.5 for 16 h. Cells were stained with LIVE/DEAD at 405-nm excitation and mouse anti-VP30 18C with IgG1 cross-adsorbed goat anti-mouse Alexa Fluor 488 secondary antibody. VP30-positive cells in control and treated cells across four experimental repeats are shown. The significance of results was determined by one-way ANOVA. (B) PBMs were treated with the indicated compound at 10 μM and infected with Georgia 2007/1 at an MOI of 1.0 for 18 h. PBMs were fixed in 4% paraformaldehyde and stained with anti-VP30 (anti-mouse Alexa Fluor 555) and Hoechst 33442 nuclear stain. The number of virus-positive cells per 10 fields of view was calculated. The significance was determined by one-way ANOVA. Confocal images are representative of three independent experiments. Scale bar: 25 μm. (C) Purified PBMs were treated with compound 1a or 1b at 10 μM and infected with Georgia 2007/1 at an MOI of 3.0. Cell lysates harvested 4 h postinfection were separated by SDS-PAGE, and Western blotting was used to ascertain viral VP30 protein levels normalized to β-actin. The significance was determined by one-way ANOVA, relative to the corresponding control. Blots representative of three independent experiments are shown. Values represent means and standard deviations. *, *P* ≤ 0.05; **, *P* ≤ 0.01; ***, *P* ≤ 0.001; ****, *P* ≤ 0.0001.

To examine if the effects of the compounds on the transcriptional level were reflected at the protein level, we assessed levels of early and late proteins following treatment with compounds 1a and 1b and infection with Georgia 2007/1 in primary porcine purified PBMs. First, we examined levels of VP30, encoded by *CP204L*, by Western blotting, immunofluorescence confocal microscopy, and flow cytometry at the early stages of infection (4 h postinfection) and late stages of infection (16 to 18 h postinfection). The results showed that compound 1a and 1b treatment did not significantly reduce VP30-positive cells following infection with Georgia 2007/1, determined by flow cytometry in a 16-h infection or by confocal microscopy in an 18-h infection (Fig. 6A and B). In addition, there was no significant reduction in VP30 levels in compound 1a- or 1b-treated cells relative to the control cells at 4 h postinfection, suggesting that the slight changes in gene expression are not reflected at the protein level (Fig. 6C).

**FIG 7:**
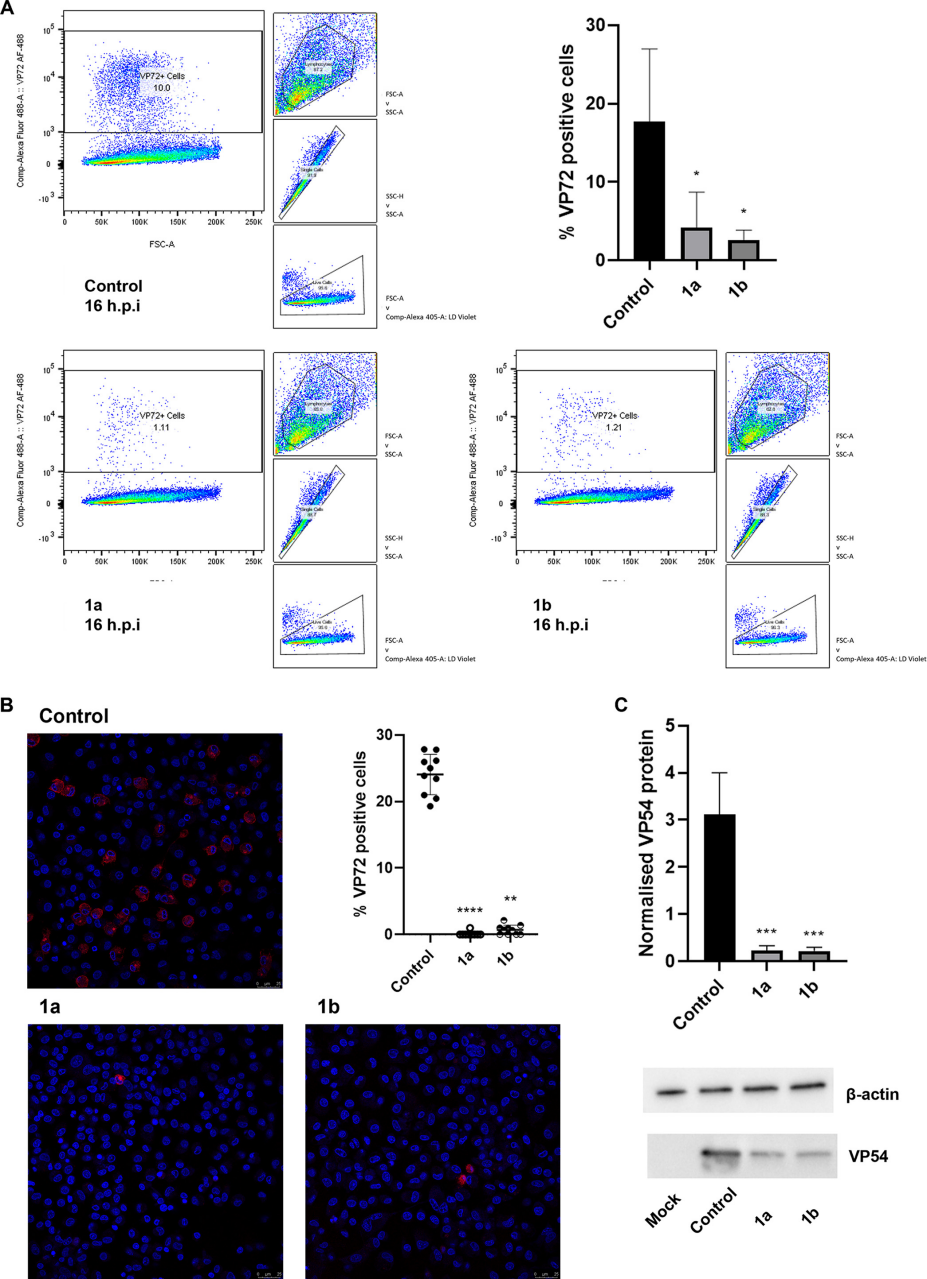
Reduction in late protein expression following treatment with compound 1a or 1b. (A) Purified PBMs were treated with compound 1a or 1b at 10 μM or DMSO and infected with Georgia 2007/1 at an MOI of 0.5 for 16 h. Cells were stained with LIVE/DEAD at 405-nm excitation and mouse anti-VP72 4H3 with IgG2a cross-adsorbed goat anti-mouse, Alexa Fluor 488 secondary antibody. VP72-positive cells in control and treated cells across four experimental repeats are shown. Significance was determined by one-way ANOVA. (B) PBMs were treated with the indicated compound at 10 μM and infected with Georgia 2007/1 at an MOI of 1.0 for 18 h. PBMs were fixed in 4% paraformaldehyde and stained with anti-VP72 (anti-mouse Alexa Fluor 555) and Hoechst 33442 nuclear stain. The number of virus positive cells per 10 fields of view was calculated. The significance was determined by Kruskal-Wallis test. Confocal images representative of three independent experiments. Scale bar: 25 μm. (C) Purified PBMs were treated with compound 1a or 1b at 10 μM and infected with Georgia 2007/1 at an MOI of 3.0. Protein lysate harvested 18 h postinfection was used to ascertain viral P54 protein levels normalized to β-actin. Significance was determined by one-way ANOVA, relative to the corresponding control. Blots are representative of three independent experiments. Values represent means and standard deviations. *, *P* ≤ 0.05; **, *P* ≤ 0.01; ***, *P* ≤ 0.001; ****, *P* ≤ 0.0001.

However, compounds 1a and 1b displayed a significant inhibitory effect on late protein levels, demonstrated by a significant reduction in VP72-positive cells (Fig. 7A and B) and a significant reduction in levels of VP54, a late protein encoded by *E183L*, at the later stages of ASFV replication (16 to 18 h postinfection) (Fig. 7C).

These data suggest that the reductions in late gene expression are reflected at the protein level and that compounds 1a and 1b mediated inhibition of late but not early gene expression.

### Delayed addition of amidate prodrug 1b after DNA replication abrogates its antiviral activity.

**FIG 8:**
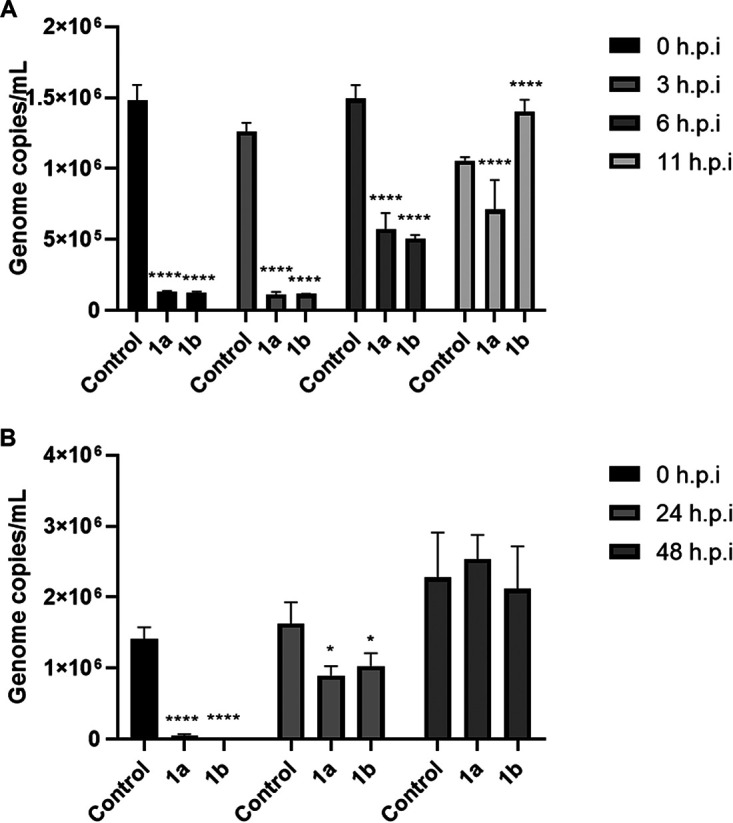
Delayed addition of phosphonamidate nucleoside prodrugs 1a and 1b. (A) Purified PBMs were infected with Georgia WT at an MOI of 0.25. The cells were incubated at 4°C for 90 min to synchronize infection. Compound 1a or 1b was added postsynchronization at the indicated time points at a final concentration of 5 μM. Supernatants were collected 24 h after infection and DNA was extracted to measure levels of virus genome by real-time PCR. (B) Purified PBMs were infected with Georgia WT at an MOI of 0.1. Compound 1a or 1b or an equivalent volume of DMSO was added immediately postinfection (0 h) or at 24 h or 48 h postinfection at a final concentration of 1 μM. The supernatants were collected 72 h after infection for DNA extraction. Viral genome copies in the supernatant were determined by real-time PCR, relative to a VP72 plasmid standard curve. Graphs are representative of 3 independent experiments. Values represent means and standard deviations. Significance was determined by one-way ANOVA, relative to the corresponding control. *, *P* ≤ 0.05; **, *P* ≤ 0.01; ***, *P* ≤ 0.001; ****, *P* ≤ 0.0001.

Finally, we conducted a time of addition assay to identify the point of inhibition by compounds 1a and 1b on ASFV replication. The infection was synchronized by incubating the cells immediately after infection at 4°C for 1.5 h, and the compounds were subsequently added immediately after synchronization (0 h) or at 3 h, 6 h, or 11 h postsynchronization. Levels of virus replication were determined by measuring genome copy numbers in cell supernatants by real-time qPCR. Both compounds were most effective when added during the early stages of ASFV replication between 0 and 6 h postinfection. Interestingly, despite the similarities from the previous data, compound 1a retained its antiviral activity at all time points, while compound 1b did not significantly inhibit ASFV replication when added at 11 h postinfection, after DNA replication (Fig. 8A). Additionally, we examined the antiviral activity of the compounds when added at different time points in a 72-h infection at an MOI of 0.1. Compounds 1a and 1b significantly inhibited ASFV replication in primary cells when added immediately (0 h postinfection) and at 24 h postinfection but did not significantly inhibit progeny virus output when added at 48 h postinfection (Fig. 8B).

## DISCUSSION

Development of a safe and effective vaccine against ASFV is still in progress (31, 32). Strategies to prevent the spread of ASFV, including culling of infected herds and strict biosecurity measures, have been insufficient in many countries. This is especially true when infected wildlife reservoirs, including wild boar, are present. There is a pressing need for additional control methods, highlighted by the introduction of ASFV to China in 2018 and its rapid spread to neighboring countries (11, 33). To this end, the development of antiviral agents for viral infections of livestock has significant potential (34, 35). Antiviral agents could be an invaluable tool for combating ASFV outbreaks in the absence of a vaccine and may be used in conjunction with a vaccine to bridge the so-called “immunity gap” before vaccines become effective (35).

We evaluated the anti-ASFV activity of two *O*-alkylated acyclic nucleoside phosphonamidate prodrugs (compounds 1a and 1b) in primary porcine cells against four genotypes from the four proposed clades A, B, C, and D (36). These nucleotide analogues derive from a common parent compound, (*R*)-*O*-2-alkylated FPMPC, and differ only by their amino acid prodrug moieties (l-aspartate and valine, respectively). Amidate prodrugs of *O*-2-alkylated pyrimidine acyclic nucleosides have been found to display antiviral activity against herpesviruses, including human cytomegalovirus (HCMV), varicella-zoster virus (VZV), and hepatitis B virus (HBV) (29). We demonstrated that both compounds displayed pan-genotype antiviral activity at noncytotoxic concentrations as well as possessing high selectivity indices against each of the ASFV genotypes. The broad inhibitory activity of the compounds against multiple, distinct ASFV genotypes is a potential advantage of antiviral agents over vaccines, which may not offer cross protection against all ASFV genotypes (37).

We sought to identify the stages of viral replication inhibited by compounds 1a and 1b. Given the distinct temporally expressed classes of ASFV transcripts, we examined ASFV gene expression at different time points postinfection at both the transcript and protein levels. Since early genes are expressed prior to ASFV genome replication and late gene expression occurs after DNA replication, we could determine if the compounds inhibited the early stages of viral replication prior to DNA replication or the late stages after viral DNA replication (30). Compounds 1a and 1b were found to inhibit the late stages of ASFV replication, with a significant reduction in late gene transcripts and late protein levels observed. Expression of late gene *B646L* was inhibited by both compounds at 8 and 18 h postinfection in cells infected with Georgia 2007/1 or BOT 1/99. However, while transcript levels of early and late gene *CP204L* and early genes *I73R* and *CP312R* at 18 h postinfection displayed a reduction similar to that of *B646L*, early transcript levels were broadly unaffected at 8 h postinfection. This may be attributed to stability of the early mRNAs, thereby masking a potential inhibition of their transcription by the compounds at 8 h (38, 39). The reduction in late gene transcription was reflected at the protein level, with a significant reduction in late VP54 protein levels, determined by Western blotting, and a significant reduction in late VP72-positive cells recorded by immunofluorescence confocal microscopy and flow cytometry at 16 to 18 h postinfection. In contrast, we observed no significant reduction in early protein VP30 levels measured by Western blotting at 4 h postinfection and no reduction in VP30-positive cells at 16 to 18 h postinfection by confocal microscopy or flow cytometry.

However, despite the similarities displayed by compounds 1a and 1b, time-of-addition assays revealed a potential key difference in their mechanism of action. The reduced antiviral activity of compound 1b when added at 11 h postinfection, when DNA replication is well advanced, suggests that compound 1b may target viral genome replication. Whether this may occur by either direct inhibition of the DNA polymerase or inhibition of associated factors, such as the viral topoisomerase, remains to be determined (21, 22). In contrast, compound 1a retained its antiviral activity when added at all time points. Promoter specificity of the ASFV RNA polymerase (RNAP) for early or late gene expression may be dependent on association with specific transcription factors. We postulate that compound 1a may specifically interfere with the late ASFV-RNAP transcription complex, thereby accounting for its effect on late but not early viral transcription and its retention of antiviral activity in the time-of-addition assay (38, 39). The difference in antiviral activity observed for the l-aspartate- and valine-containing prodrugs might be attributed to a diverse cell penetration ability and/or variations in the kinetics of intracellular metabolism responsible for delivering their active phosphorylated forms. A 72-h time-of-addition assay revealed that the compounds were most effective when added up to 24 h postinfection. This suggests that the compounds may have potential use as prophylactic drugs or where infection with ASFV is rapidly identified. However, these findings may not be representative of postinfection treatment *in vivo* and do not preclude the therapeutic potential of compounds 1a and 1b in established infections *in vivo*.

In conclusion, the potent pan-genotype antiviral activity of compounds 1a and 1b combined with their tolerability, as indicated by their high selectivity indices, suggests that these compounds merit further evaluation as potential prophylactic and/or therapeutic agents against ASFV.

## MATERIALS AND METHODS

### Acyclic nucleoside phosphonamidate prodrugs.

*O*-2-Alkylated acyclic nucleoside phosphonamidate prodrugs 1a and 1b were prepared according to a previously established synthetic procedure and supplied as a 10 mM solution in dimethyl sulfoxide (DMSO) (29).

### Cell culture.

Purified pig bone marrow cells (PBMs) were isolated from bone marrow from the leg bones of outbred pigs, using Histopaque 1077 gradients (Sigma-Aldrich). PBMs were cultured in RPMI medium (Sigma-Aldrich) supplemented with 10% heat-inactivated fetal calf serum (FCS), 100 IU/mL of penicillin, 100 μg/mL of streptomycin, and 100 ng/mL of recombinant porcine macrophage colony-stimulating factor (CSF-1; Roslin Technologies) for 3 days.

### Cell viability assay.

Purified primary porcine PBMs were incubated with the antiviral compounds at the indicated serial dilutions for 72 h. Cell viability in live cells was determined using the RealTime-Glo MT cell viability assay (Promega), according to the manufacturer’s instructions for endpoint assay.

### Virus infection and titration.

Wild-type viruses Georgia 2007/1, BOT 1/99, Magadi w/hog 9, and KAB6/2 used in this study have been previously described (5, 40). Virus titers were determined on PBMs, cultured for 3 days prior to infection in Earle’s Balanced Salt Solution (EBSS) (Sigma), supplemented with 4 mM HEPES, 10% heat-inactivated porcine serum (BioSera), 100 IU/mL of penicillin, and 100 μg/mL of streptomycin at 37°C and 5% CO_2_. Virus titration was carried out by limiting dilution in PBMs, and infected cells were identified by hemadsorption in the presence of red blood cells. Virus titers were calculated using the Spearman-Karber method (41).

### Western blotting.

Cells were lysed by radioimmunoprecipitation assay (RIPA) buffer (Santa Cruz) supplemented with Pierce protease and phosphatase inhibitor minitablets (Thermo Fisher). Five micrograms of protein, concentration determined by Coomassie protein assay reagent (Thermo Fisher), was loaded to each well of 4 to 15% protein gels (Bio-Rad). Proteins were transferred onto 0.45-μm polyvinylidene difluoride (PVDF) membranes (GE Healthcare). PVDF membranes were incubated with anti-ASFV VP30 C18 primary antibody (42), anti-P54 serum, or anti-β-actin ab8227 (Abcam) at 4°C overnight. The membranes were incubated for 1 h at room temperature (RT) with horseradish peroxidase (HRP)-conjugated goat anti-mouse immunoglobulins (Agilent) or ab6721 HRP-conjugated goat anti-rabbit (Abcam) at the manufacturers’ recommended dilutions. Protein bands were detected using Pierce ECL Western blotting substrate (Thermo Fisher). The bands were semiquantified with ImageJ 1.x (43) and normalized to housekeeping protein β-actin.

### Real-time qPCR.

Total RNA was extracted from cells using the RNeasy Plus minikit (Qiagen). cDNA was synthesized from 0.5 μg of total RNA using the Superscript III first-strand synthesis kit (Invitrogen). Transcripts were evaluated by qPCR on a Stratagene Mx3005P system (Agilent Technologies) using a thermal profile of 95°C for 10 min and 40 cycles of 95°C for 15 s and 60°C for 60 s. Viral gene expression *CP204L* [F5′-TCTTTTGTGCAAGCATATACAGCTT-3′ and R5′-TGCACATCCTCCTTTGAAACAT-3′] and *B646L* [F5′-ACGGCGCCCTCTAAAGGT-3′ and R5′-CATGGTCAGCTTCAAACGTTTC-3′] was normalized to the glyceraldehyde-3-phosphate dehydrogenase gene (*GAPDH*; F5′-TCAACGACCACTTTGTCAAGC-3′ and R5′-TAAGAGCCCCTGGACCACCA-3′) and analyzed using the delta delta threshold cycle (ΔΔ*C_T_*) method. Viral DNA was extracted from the cell culture supernatant using the viral RNA minikit (Qiagen), which also allows for isolation of viral DNA from supernatant, for quantitative analysis of virus genome copy number. qPCR was carried out on a Stratagene Mx3005P system, using a modified protocol previously described, using primers VP72_Fwd (5′-CTGCTCATGGTATCAATCTTATCGA-3′) and VP72_Rev 5′-GATACCACAAGATC(AG)GCCGT-3′ and the probe 5′-(6-carboxyfluorescein [FAM])-CCACGGGAGGAATACCAACCCAGTG-3′-(6-carboxytetramethylrhodamine [TAMRA]) and a thermal profile of 95°C for 5 min and 40 cycles of 95°C for 15 s and 60°C for 30 s (44).

### Confocal microscopy.

ASFV-infected cells were fixed in 4% paraformaldehyde at 37°C for 30 min and RT for 30 min. The cells were permeabilized with 0.1% Triton X-100 for 15 min at RT. The cells were blocked in 3% bovine serum albumin (BSA) in phosphate-buffered saline (PBS) for 1 h at RT before incubation with the indicated primary antibody, anti-ASFV VP72 monoclonal antibody 4H3 (45) or VP30 C18, for 1 h at RT. The cells were washed thrice with PBS before incubation with secondary antibody Alexa Fluor 568-conjugated anti-mouse (Abcam), diluted 1:500, for 1 h at RT. Nucleic acid staining was done with 5 μg/mL of Hoechst 33342 for 15 min at RT. Imaging was performed using a Leica TCS SP8 confocal microscope using a 40× oil immersion objective.

### Flow cytometry.

Purified PBMs, infected with Georgia 2007/1 at an MOI of 1.0 for the indicated duration, were suspended in PBS and stained with the Molecular Probes LIVE/DEAD fixable violet dead cell stain kit at 405-nm excitation (Thermo Fisher), according to the manufacturer’s instructions. The cells were washed twice with PBS and fixed in 4% paraformaldehyde (PFA) for 1 h. The cells were permeabilized in 0.2% saponin (Sigma-Aldrich) and 0.5% BSA in PBS for 10 min at RT. ASFV proteins were labeled with anti-ASFV VP72 monoclonal antibody 4H3 or VP30 C18 at 1:500 in the permeabilization solution for 30 min at RT. The cells were subsequently incubated with secondary antibodies IgG2a cross-adsorbed goat anti-mouse, Alexa Fluor 488 or IgG1 cross-adsorbed goat anti-mouse, and Alexa Fluor 488 (Thermo Fisher) at 2 μg/mL for 30 min at RT. The stained cells were suspended in 400 μL of PBS in fluorescence-activated cell sorter (FACS) tubes (BD Biosciences) for subsequent analysis by flow cytometry. Data were collected on an LSR Fortessa cell analyzer (BD Biosciences) and analyzed with FlowJo 10.7.1 software. Cell debris was removed from the analysis by gating cell populations using forward and side scatter area; doublets were removed by selecting single cell populations using side scatter height versus area and VP30- or VP72-positive cells were identified in the live cell population. The percentages of VP30- and VP72-positive cells were compared in control cells and cells treated with compound 1a or 1b.

### Statistical analysis.

Statistical analysis was performed with GraphPad Prism version 8.1.2. Linear regression analysis on normalized values was used to determine the 50% cytotoxic concentration (CC_50_) and the 50% inhibitory concentration (IC_50_). The data were analyzed by Dunnett’s multiple-comparison test (two-way analysis of variance [ANOVA]) and one-way ANOVA, as described in the figure legends. The data are presented as means ± standard errors. At least three independent experiments were performed; representative results are presented. A *P* value of <0.05 was considered significant.
